# Prophylactic Low-Dose Paracetamol Administration for Ductal Closure and Amplitude-Integrated Electroencephalography in Preterm Infants

**DOI:** 10.3389/fped.2022.887614

**Published:** 2022-05-23

**Authors:** Christina Schreiner, Maria Sappler, Michaela Höck, Marlene Hammerl, Vera Neubauer, Ursula Kiechl-Kohlendorfer, Elke Griesmaier

**Affiliations:** Department of Pediatrics II (Neonatology), Medical University of Innsbruck, Innsbruck, Austria

**Keywords:** ductal closure, preterm infant, amplitude-integrated electroencephalography, neurodevelopmental outcome, paracetamol

## Abstract

**Introduction:**

Prophylactic low-dose paracetamol administration is used to induce closure of the ductus arteriosus in preterm infants. In our recent study we found no impairment on microstructural maturation processes in the brain of preterm infants at term-equivalent age following prophylactic low-dose paracetamol administration. We now assessed amplitude-integrated electroencephalography (aEEG) signals in preterm infants with and without exposure to prophylactic low-dose paracetamol administration.

**Methods:**

Infants <32 gestational weeks born between 10/2014 and 12/2018 received prophylactic paracetamol (10 mg/kg intravenously every 8 h until echocardiography after at least 72 h) and form the paracetamol group; infants born between 02/2011 and 09/2014 formed the control group. Four single parameters (continuity, cyclicity, amplitude of lower border, bandwidth span) together with their sum (Burdjalov total score) and presence of sleep-wake cycles were compared between the groups.

**Results:**

Included in the study were 338 infants. Two-hundred and seventeen infants received prophylactic paracetamol and 121 formed the control group. The paracetamol group showed a significantly higher number of sleep-wake cycles per hour and a significantly higher total scores compared to the control group (*p* < 0.05).

**Conclusion:**

Paracetamol exposure has been regarded critically with respect to safety in preterm infants in recent years. We found no impairment on amplitude-integrated electroencephalography signals in preterm infants receiving low-dose prophylactic paracetamol compared to controls. Growing awareness and greater availability of data may encourage the clinicians to administer prophylactic paracetamol for ductal closure in preterm infants. The clinical relevance of our findings has to be evaluated in long-term follow up studies on neurodevelopmental outcome.

## Introduction

Patent ductus arteriosus (PDA) is observed in more than 30% of preterm infants with a gestational age of 32 weeks (1). Cyclooxygenase inhibitors are the therapeutics of choice to induce ductal closure in preterm infants, but they also carry substantial risks that may outweigh the benefit (2–4). Despite a large body of research, there is controversy about the management of ductal closure in preterm infants (5). In the last years, the management of PDA changed from pharmacologic and/or surgical intervention to a less aggressive approach and rather watchful wait and see strategy (6). Paracetamol showed promising results as pharmacological option for ductal closure, with fewer short-term side-effects than have been reported for cyclooxygenase inhibitors, but concerns about its effect on brain development and function have been raised (7–11).

To shed some more light on the ongoing debate about the safety of prophylactic low-dose paracetamol administration and preterm brain development, we investigated its effect in a large cohort of very preterm infants. First we used magnetic resonance-based diffusion tensor imaging at term-equivalent age in preterm infants with and without exposure to prophylactic low-dose paracetamol and found no impairment of microstructural maturation processes (12).

The aim of the current study was to investigate the effect of prophylactic low-dose paracetamol administration on functional brain development in this large cohort of preterm infants. We analyzed amplitude-integrated electroencephalography (aEEG) signals in the first 4 weeks of life in very preterm infants with and without exposure to prophylactic low-dose paracetamol.

## Materials and Methods

### Study Population and Study Design

**FIGURE 1:**
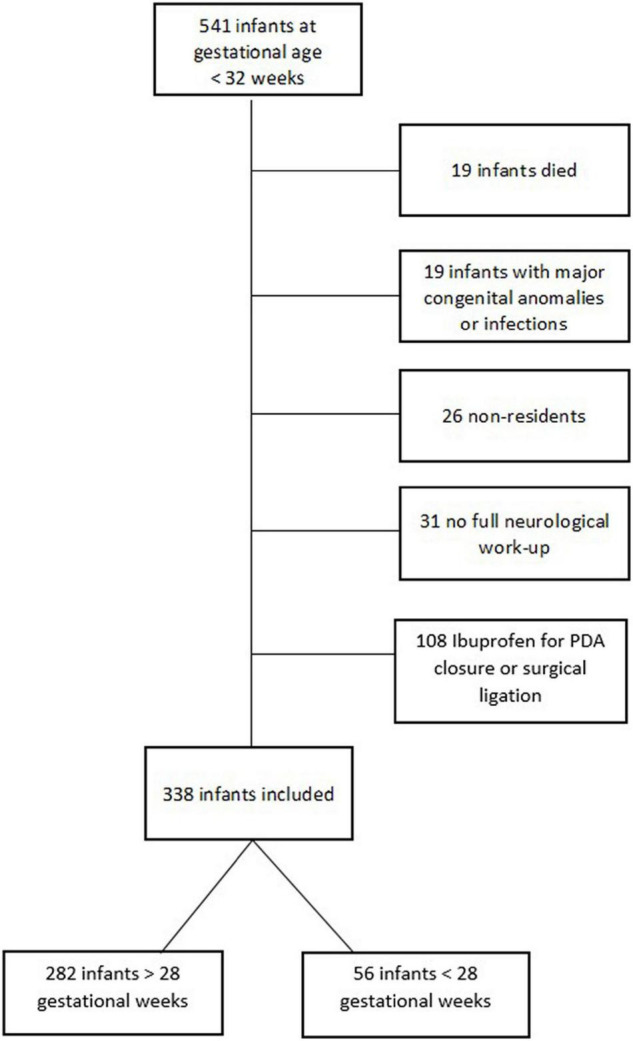
Flowchart of the inclusion and exclusion procedures.

This study was conducted as a retrospective analysis of prospectively collected data. Infants born at less than 32 weeks of gestation between February 2011 and December 2018 at Innsbruck Medical University were enrolled. The detailed inclusion and exclusion criteria are shown in Figure 1. Maternal and neonatal data were collected during the hospital stay and described in our previous work (13). Detailed information concerning echocardiographic assessment and PDA treatment has been published in detail in our recent paper.

### Paracetamol Administration

In October 2014, the administration of prophylactic low-dose paracetamol was introduced in our department for all infants born at less than 32 gestational weeks. Infants born until September 2014 were included in the control group and infants born after September 2014 in the paracetamol group. Infants in the paracetamol group received prophylactic low-dose paracetamol (Perfalgan©, Bristol-Meyers Squibb; 10 mg/kg birthweight intravenously, every 8 h) within the first day of life without reference to the state of the ductus until routine echocardiography was performed, after at least 72 h of life. Infants in the control group received no preventive therapy. Each paracetamol administration in the control group and the administrations in addition to the low-dose paracetamol prophylaxis in the paracetamol group are summarized and referred to as “additional paracetamol.” Specifically, throughout the study period paracetamol was routinely used during ophthalmologic examinations in a dosage of 20 mg/kg body weight given orally and as analgesic drug peri-operatively.

### Amplitude-Integrated Electroencephalography Recording and Assessment Details

Recording of aEEG during the first 4 weeks of life is part of our routine program for all preterm infants born at less than 32 gestational weeks (14). Recordings were acquired with the BrainZ instruments BRM 3 monitor (Natus Medical Inc., San Carlos, CA, United States) from four electrodes placed at the C3, C4, P3, and P4 positions according to the international 10–20 system of electrode placement modified for neonates. Using the P3 and P4 electrodes which are positioned parietal, a cross cerebral recording was computed. Signals were amplified, filtered, smoothened, and time-compressed and displayed on a semilogarithmic scale at a speed of 6 centimeters per hour. Traces with impedance >10 kOhm, with obvious or marked artifacts or recordings that were conducted under sedative medication were omitted from analysis. According to our standard protocol aEEG was recorded during the first 72 h of life and weekly from postnatal week one to week four. Recordings were evaluated by one experienced operator (CS) blinded to clinical data, and in the case of suspected artifacts, discussed with a second experienced observer and consensus was reached (13). We chose defined time periods, each of a duration for 6 h for assessment (18–24 h on day 1, 42–48 h on day 2, and 66–72 h on day 3). For the weekly recordings we evaluated the longest section of tracings for assessment (3–6 h) as described in detail previously (15).

The aEEG recordings were evaluated for

1. Background scoring, according to the method described by Burdjalov et al., including four single parameters: continuity (0–2 points), cycling (0–5 points), amplitude of the lower border (0–2 points), bandwidth span combined with the amplitude of the lower border (0–4 points). The sum equals the total score (maximum of 13 points) (16).
2. Presence of sleep-wake cycles as described by Olischar et al. (17).

#### Statistical Analysis

Data are presented as numbers (frequencies;%), means with standard deviation or 95% confidence intervals and medians with 25th and 75th centiles. The Mann–Whitney *U* test, Student’s *t*-test, and the χ2 test were used where appropriate. Multivariate logistic regression analysis including significant differences in patient characteristics (gestational age, invasive ventilation) was used to account for potential confounders, presented as odds ratio (OR) and 95% confidence interval (CI). A *p*-value < 0.05 was considered statistically significant. Data analysis was performed using SPSS, version 24.0 for Windows (IBM Corp., Armonk, NY, United States). Boxplots were generated using the software R, version 3.6.3.

## Results

### Study Population

A total of 338 preterm infants between 24 + 0 and 31 + 6 gestational weeks were included, of whom 217 (64.2%) patients received early low-dose prophylactic paracetamol administration (paracetamol group) and 121 (35.8%) patients formed the control group. Referring to our recent work on this study cohort, we excluded two infants from analysis, who did not receive an aEEG recording. Maternal characteristics were equally distributed among the groups. Infants of the paracetamol group were born at a significantly lower median gestational age than were the controls (29.9 weeks vs. 30.7 weeks, *p* = 0.020). There was a difference in the frequency of invasive ventilation, with infants in the paracetamol group being less often ventilated (44.7% vs. 58%; *p* = 0.020). All other variables were similar between the groups (*p* > 0.05) (Table 1).

**TABLE 1:** Patients’ characteristics.

Variable	Paracetamol group *n* = 217	Control group *n* = 121	*p* value*	Paracetamol group *n* = 39	Control group *n* = 17	*p* value*
Gestational age (weeks) (median, IQR)	29.9 (28.5; 31.2)	30.7 (29.0; 31.4)	**0.020** ^‡^	26.7 (25.7; 27.4)	26.9 (25.6; 27.4)	0.979^‡^
Gestational age below 28 weeks (*n*, %)	39 (18.0)	17 (14.0)	0.352^a^			
Birth weight (grams) (mean, SD)	1296 ± 351	1361 ± 408	0.142°	869 ± 208	799 ± 198	0.377^‡^
Small for gestational age (*n*, %)	13 (6.0)	11 (9.1)	0.287^a^	3 (7.7)	4 (23.5)	0.099^a^
Male (*n*, %)	126 (58.1)	59 (48.8)	0.099^a^	22 (56.4)	8 (47.1)	0.519^a^
Antenatal steroids (*n*, %)	200 (92.2)	114 (95.0)	0.323^a^	37 (94.9)	16 (94.1)	0.908^a^
Premature rupture of membranes > 24 h (*n*, %)	47 (21.7)	21 (17.9)	0.365^a^	9 (23.1)	7 (41.2)	0.168^a^
Apgar Score 5 min < 7 (*n*, %)	13 (6.0)	10 (8.3)	0.440^a^	2 (5.1)	7 (41.2)	<0.001^a^
Surfactant treatment (*n*, %)	159 (74.0)	89 (73.6)	0.936^a^	36 (92.3)	16 (94.1)	0.809^a^
Invasive ventilation (*n*, %)	97 (44.7)	69 (58)	0.020^‡^	24 (61.5)	13 (76.2)	0.278^a^
Catecholamine therapy (*n*, %)	10 (4.8)	7 (5.8)	0.797^a^	3 (8.1)	2 (11.8)	0.667^a^
Early-onset sepsis (*n*, %)	15 (6.9)	4 (3.4)	0.178^a^	7 (17.9)	3 (18.8)	0.944^a^
Late-onset sepsis (*n*, %)	21 (9.7)	13 (10.7)	0.755^a^	10 (25.6)	8 (47.1)	0.115^a^
Bronchopulmonary dysplasia (*n*, %)	38 (17.5)	23 (19.0)	0.732^a^	28 (71.8)	15 (88.2)	0.180^a^
Necrotizing enterocolitis (*n*, %)	6 (2.8)	3 (2.5)	0.833^a^	2 (5.4)	1 (5.9)	0.943^a^
Patent ductus arteriosus (*n*, %)	30 (13.8)	12 (9.9)	0.296^a^	9 (23.1)	4 (23.5)	0.971^a^
Retinopathy of prematurity grade 3–4 (*n*, %)	3 (1.5)	6 (5.0)	0.062^a^	1 (2.7)	2 (11.8)	0.177^a^
Intracranial hemorrhage any grade (*n*, %)	29 (13.4)	12 (9.9)	0.390^a^	8 (20.5)	4 (23.5)	0.800^a^
Intracranial hemorrhage grade 3 or 4 (*n*, %)	3 (1.4)	3 (2.5)	0.464^a^	0	1 (5.9)	0.126^a^

Patients’ characteristics of the study cohort and by group and for infants born below 28 weeks gestational age with n (%), median (IQR = interquartile range), and mean (± standard deviation), *paracetamol vs. no paracetamol; P values are from the ^a^χ2 test, Student’s t-test, and ^‡^Mann-Whitney U test, as appropriate.

*In all variables the proportion of missing data was <5%. Bold means p < 0.05.*

### Paracetamol Administration

Preterm infants in the paracetamol group received a median of total 109 mg/kg (90–130) of paracetamol as early low-dose prophylaxis. The median amount of paracetamol administered in addition to the early low-dose prophylactic paracetamol at any time during the hospital stay was 21 mg/kg (20–60) and did not differ significantly from the control group receiving 21 mg/kg (20–40), (*p* > 0.15).

### Amplitude-Integrated Electroencephalography Signals in the Neonatal Period

**FIGURE 2:**
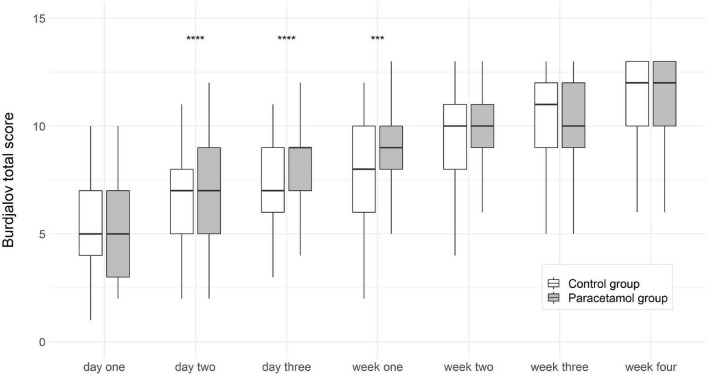
Boxplots of Burdjalov total score (median, interquartile range) at each time point in the paracetamol and control group. Significant results of the logistic regression are marked with asterisks. ***p < 0.001, ****p < 0.0001.

**FIGURE 3:**
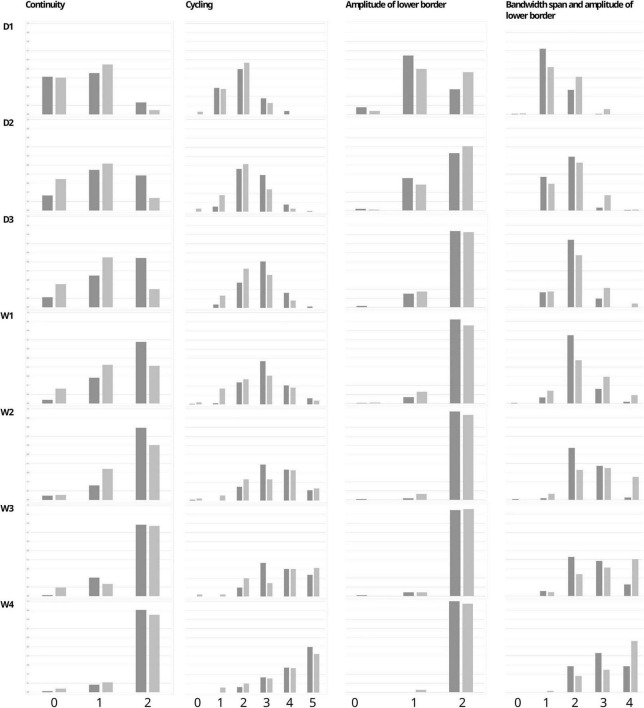
Percentage of infants with each Burdjalov single score (Continuity, Cycling, Amplitude of the lower border, and the Bandwidth span and amplitude of lower border) at each time point in the paracetamol and control group.

Comparison of the two groups showed significant differences in the Burdjalov total score and separate scores. In the paracetamol group as compared to the control group there were significantly higher total scores at day 2 [7 (2–12) vs. 7 (2–11); *p* = 0.032], day 3 [9 (3–12) vs. 7 (3–11); *p* = 0.002], and week 1 [9 (1–13) vs. 8 (2–12); *p* = 0.027]. After adjustment for gestational age and invasive ventilation consistent results were shown (*p* < 0.001) (Figure 2). The separate scores are shown in Figure 3.

**FIGURE 4:**
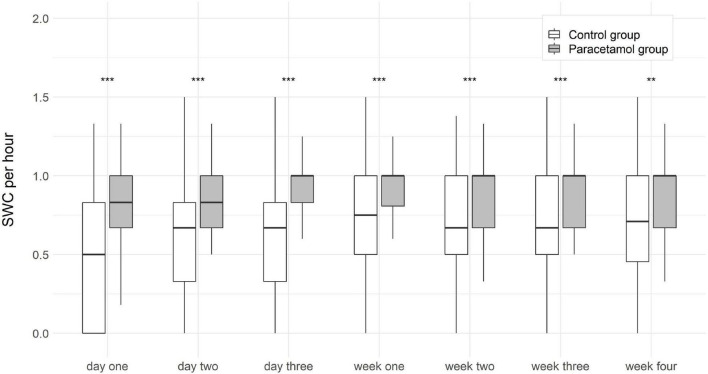
Boxplots of sleep-wake cycles (SWC) per hour at each time point in the paracetamol and control group. Significant results of the logistic regression are marked with asterisk. **p < 0.01, ***p < 0.001.

In order to better quantify differences in both amplitude and continuity of electrical activity, conferring to sleep-wake cycles in aEEG traces, we also assessed the absolute number of sleep-wake cycles. The number of sleep-wake cycles per hour were significantly higher in the paracetamol group as compared to the control group at day 1 [0.8 (0.0–1.5) vs. 0.5 (0.0–1.3); *p* < 0.001], day 2 [0.83 (0–1.5) vs. 0.7 (0.0–1.5); *p* < 0.001], day 3 [1.0 (0.3–2) vs. 0.7 (0.0–2.0); *p* < 0.001], week 1 [1.0 (0–1.67) vs. 0.7 (0.0–2.0); *p* < 0.001], week 2 [1.0 (0.3–1.5) vs. 0.7 (0–1.4); *p* < 0.001], week 3 [1.0 (0–1.5) vs. 0.7 (0.0–2.0); *p* < 0.001], and week 4 [1.0 (0.3–3.0) vs. 0.7 (0.0–2.0); *p* = 0.002]. After adjustment for gestational age and invasive ventilation the differences were still statistically significant (Figure 4).

### Infants Born <28 Gestational Weeks

For infants born at less than 28 gestational weeks a subgroup analysis was performed. Regarding maternal and neonatal characteristics given in Table 1, infants in the control group showed an Apgar score below 7 at 5 min more frequently than infants in the paracetamol group (41.2% vs. 5.1%; *p* < 0.001). All other variables did not differ significantly. Fifty-six preterm infants were analyzed, 39 infants (69.6%) formed the paracetamol group and 17 infants (30.4%) the control group.

**FIGURE 5:**
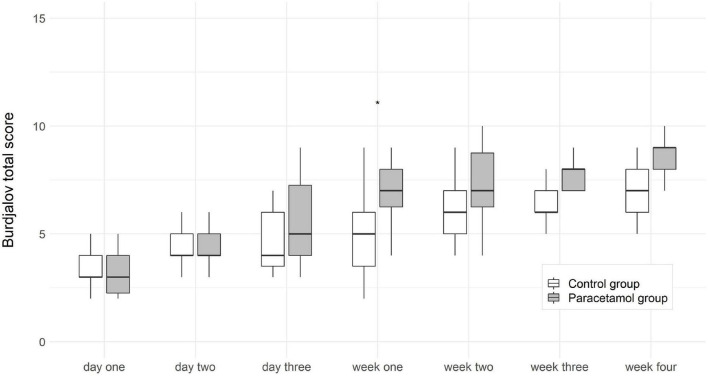
Boxplots of Burdjalov total scores (median, interquartile range) at each time point in the paracetamol and control group for infants born <28 gestational weeks. Significant result of the logistic regression is marked with asterisk. *p < 0.05.

**FIGURE 6:**
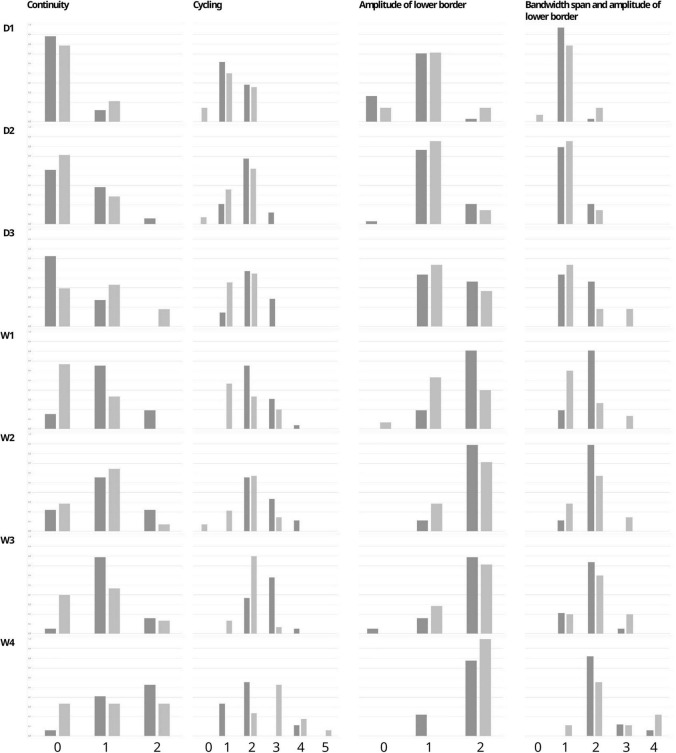
Percentage of infants with each Burdjalov single score (Continuity, Cycling, Amplitude of the lower border, and the Bandwidth span and amplitude of lower border) at each time point in the paracetamol and control group for infants born <28 gestational weeks.

Group comparisons showed higher total scores in infants in the paracetamol group at week 1 [7 (4–9) vs. 5 (2–9); *p* = 0.001], week 3 [8 (3–10) vs. 6 (4–10); *p* = 0.035], and week 4 [9 (6–13) vs. 7 (5–12); *p* = 0.038]. After adjustment for the Apgar score below 7 at 5 min the difference at week 1 remained significant (*p* = 0.010) (Figure 5). The separate scores are shown in Figure 6.

**FIGURE 7:**
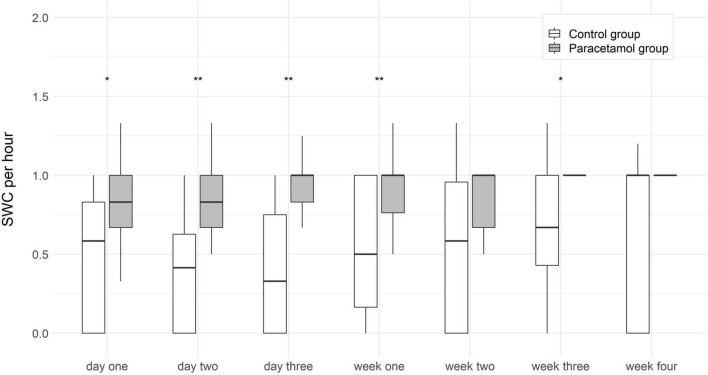
Boxplots of sleep-wake cycles per hour at each time point in the paracetamol and control group for infants born <28 gestational weeks. Significant results of the logistic regression are marked with asterisk. **p < 0.01, *p < 0.05.

The number of sleep-wake cycles per hour were significantly higher in the paracetamol group at day 1 [0.8 (0.1–1.5) vs. 0.6 (0.0–1.0); *p* = 0.020], day 2 [0.83 (0–1.5) vs. 0.4 (0.0–1.0); *p* < 0.001], day 3 [1.0 (0.3–2.0) vs. 0.3 (0.0–1.0); *p* = 0.002], week 1 [1.0 (0.0–1.7) vs. 0.5 (0.0–1.0); *p* = 0.004], week 2 [1.0 (0.5–1.5) vs. 0.6 (0–1.3); *p* = 0.022], and week 3 [1.0 (0.5–1.5) vs. 0.7 (0.0–1.3); *p* = 0.015]. After adjustment for the Apgar score below 7 at 5 min the differences were still statistically significant at day 1 (*p* = 0.029), day 2 (*p* = 0.004), day 3 (*p* = 0.009), week 1 (*p* = 0.010), and week 3 (*p* = 0.033). Significance was lost at week 2 (Figure 7).

## Discussion

Patent ductus arteriosus is a common cause of morbidity and mortality among preterm infants and can have serious effects on this vulnerable patient population. One treatment modality is to induce ductal closure, even before signs of a significant shunt occur. Since the first description of this prophylactic approach in 1982, studies on prophylactic treatment of PDA in preterm infants were related to the application of indomethacin and ibuprofen for a long time (18, 19). Forty years later, prophylactic use of cyclooxygenase inhibitors is still regarded with caution, bearing in mind the high prevalence of side effects in this vulnerable preterm population (20). During the last years several studies have been published on prophylactic paracetamol use for ductal closure in preterm infants, showing promising results (11, 21, 22). Despite years of research and clinical experience, leading to prophylactic low-dose paracetamol administration to become a routine pharmacological strategy in preterm infants, to induce ductal closure, many issues remain unresolved. In particular, the safety of paracetamol administration in preterm infants remains an area of ongoing debate.

A review of nine studies suggested a higher risk of adverse neurological outcome after fetal paracetamol exposure during pregnancy, especially for autism spectrum disorder and attention deficit hyperactivity with a dose-response gradient (10). This adverse prenatal impact might be caused by the fact that the mother produces a toxic metabolite of paracetamol (*N*-acetyl-p-benzoquinone imine), which passes the placental barrier (23, 24). A small follow-up study compared intravenous paracetamol to placebo injections in the first days of life in 44 preterm infants, they found no adverse effects at two and five years of age (25, 26). There are no further data on the impact of prophylactic paracetamol in preterm infants.

We previously confirmed a significantly lower rate of PDA following prophylactic paracetamol administration in a large cohort of preterm infants born below 32 weeks gestational age. Infants in the paracetamol group showed lower rates of bronchopulmonary dysplasia, retinopathy of prematurity and late onset sepsis. We did not detect any serious adverse effects (8). Beyond that study, we further investigated the effect of prophylactic low-dose paracetamol administration on structural and functional brain development. We found no impairment on microstructural maturation processes in preterm infants following prophylactic low-dose paracetamol administration (12). The present study is the next step in the continuation of our investigations, in which we were the first to study functional brain development in very preterm infants with and without exposure to low-dose paracetamol exposure.

The aEEG offers the possibility to monitor electrocortical activity after birth and during postnatal development at the neonatal intensive care unit. The presence of sleep-wake cycles gives information about brain function during development. The absence of sleep-wake cycles coheres with brain lesions, cerebral hemorrhages, higher CRIB scores and mortality rates in preterm infants (27, 28). A correlation between sleep-wake cycles and neurodevelopmental outcome has been shown previously (29, 30). We found higher numbers of sleep-wake cycles in the paracetamol group as compared to the control group in the first 4 weeks of life in the overall cohort, and in the first 3 weeks of life in the subgroup of extremely preterm infants.

We also analyzed another well-established parameter for brain function, which is the Burdjalov score, introduced in 2003. This scoring system assesses brain activity in term and preterm infants and encompasses four basic components of aEEG evaluation, namely, continuity, cycling, amplitude of the lower border and the bandwidth (16, 31). We found higher total scores in the paracetamol group as compared to the control group at day 2, day 3, and week 1 in the overall cohort, and at week 1 in the subgroup of extremely preterm infants.

We observed a maturational advance in the paracetamol group in the first weeks of life. One might assume, that this could be due the analgesic effect of paracetamol. In one of our previous studies, we assumed a beneficial effect on pain perception in preterm infants, receiving prophylactic low-dose paracetamol regarding less frequent administration of oral dextrose within the first week of life (8). In 2021, Lavanga et al. showed that exposure to early procedural pain in preterm infants, increased the level of discontinuity in EEG signals (32). An amelioration of painful procedures by paracetamol, seems to be a convincing explanation of the observed effect in our cohort. However, within this study, we did not systematically collect data on pain measures and number of painful procedures, to further evaluate this hypothesis.

One of the strengths of our study is the large study cohort. At our department aEEG is used as a standard tool in all preterm infants born below 32 completed weeks of gestation, thus, we serve with a representative population that did not undergo any preselection. The numerous evaluation points of aEEG provide a surveillance of the development of brain activity during neonatal life. The retrospective design of our study, with a historical cohort (control group) is a limitation. However, including changes in neonatal resuscitation (such as invasive ventilation to account for the implementation of less invasive surfactant administration) in our regression models did not change the results.

## Conclusion

We found no impairment of electrocortical signals in preterm infants following low-dose prophylactic paracetamol administration. We observed a maturational advance in the paracetamol group in the first weeks of life. We are unaware, whether these differences in functional and structural brain development, will also be reflected in neurodevelopmental outcome. Thus, we will further evaluate the clinical relevance of our findings in the long-term. We feel confident that our results are of interest for clinicians. Growing awareness and greater availability of data may further encourage prophylactic paracetamol administration for ductal closure in preterm infants.

## Data Availability Statement

All data generated or analyzed during this study are included in this article. Further inquiries can be directed to the corresponding author.

## Ethics Statement

The study was approved by the Ethics Committee of the Medical University of Innsbruck (Study No. AN2013_0086 and AN2013_0086_33_4_2). As this was a retrospective analysis, written informed consent was not required.

## Author Contributions

EG and VN initiated and designed the study. CS, MHa, MHö, and MS participated in data collection. EG, MHa, and CS sampled and evaluated aEEG recordings. CS, MS, and EG wrote the first draft and submitted the final version for publication. EG, VN, and UK-K supervised the study. All authors participated in data interpretation and contributed to drafting and revising the manuscript.

## Conflict of Interest

The authors declare that the research was conducted in the absence of any commercial or financial relationships that could be construed as a potential conflict of interest.

## Publisher’s Note

All claims expressed in this article are solely those of the authors and do not necessarily represent those of their affiliated organizations, or those of the publisher, the editors and the reviewers. Any product that may be evaluated in this article, or claim that may be made by its manufacturer, is not guaranteed or endorsed by the publisher.
